# Relationship Among *Blastocystis*, the *Firmicutes/Bacteroidetes* Ratio and Chronic Stress in Mexican University Students

**DOI:** 10.1007/s00284-021-02756-7

**Published:** 2022-01-24

**Authors:** Janeth Oliva Guangorena-Gómez, Iliana Itzel Lozano-Ochoa, Ilse Lizeth Rivera-Medina, Alejandra Méndez-Hernández, Jorge Antonio Espinosa-Fematt, Claudia Muñoz-Yáñez

**Affiliations:** Laboratory of Molecular and Cellular Microbiology and Department of Investigation Faculty of Health Sciences, Universidad Juárez del Estado de Durango, Calz. Palmas 1, Revolución, 35050 Gómez Palacio, Durango México

## Abstract

The role played by *Blastocystis* in humans has been a subject of discussion due to its intestinal effects and modifications in the intestinal microbiota. We aimed to analyze the relationship between *Blastocystis* subtypes ST1-4 and 7, the *Firmicutes* to *Bacteroidetes* ratio (*F/B* ratio) of fecal microbiota, and chronic stress in university students. This study had a cross-sectional design with a sample of 202 students. We analyzed fecal and hair samples, and stress inventories were applied to the students. The results showed a frequency of *Blastocystis*-colonized students of 52.97%. Regarding fecal microbiota, a median RAU of 0.801 for *Firmicutes* and 0.82 of *Bacteroidetes* were obtained, with an *F/B* ratio of 0.83. A low *F/B* ratio (66.04%) was more frequent in *Blastocystis*-colonized students, whereas a high *F/B* ratio (68.09%) (*p* =  < 0.0001) was found in the *Blastocystis*-non-colonized. Only *Blastocystis* ST3 did not significantly correlate with a low *F/B* ratio (*p* = 0.290). The ST4 was associated with lower values of cortisol (*p* = 0.030), psychological stress (*p* = 0.040), and lower frequency of constipation (*p* = 0.010). Only two students with the ST1 had abdominal pain (*p* = 0.007). Our results suggest that colonization by *Blastocystis* subtypes can modify the intestinal microbiota due to a decreased ratio between the two most representative phyla *(F/B).* Also, the results of this study show that ST4 colonization is related to a lower level of chronic stress.

**Graphical abstract:**

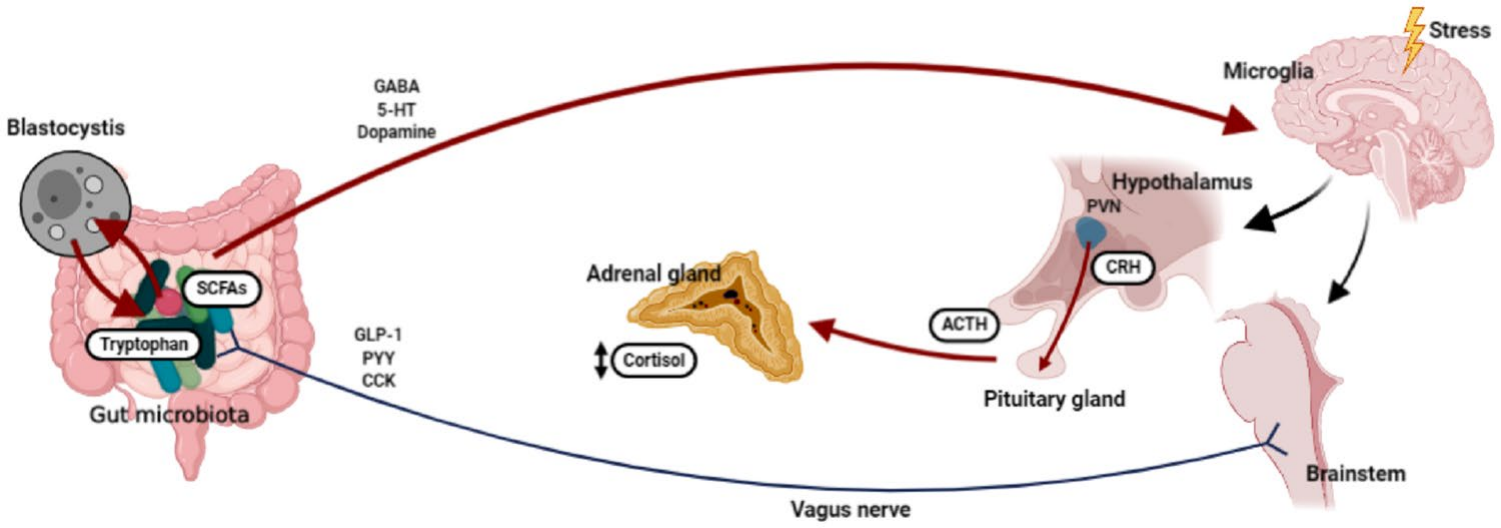

**Supplementary Information:**

The online version contains supplementary material available at 10.1007/s00284-021-02756-7.

## Introduction

**Fig. 1:**
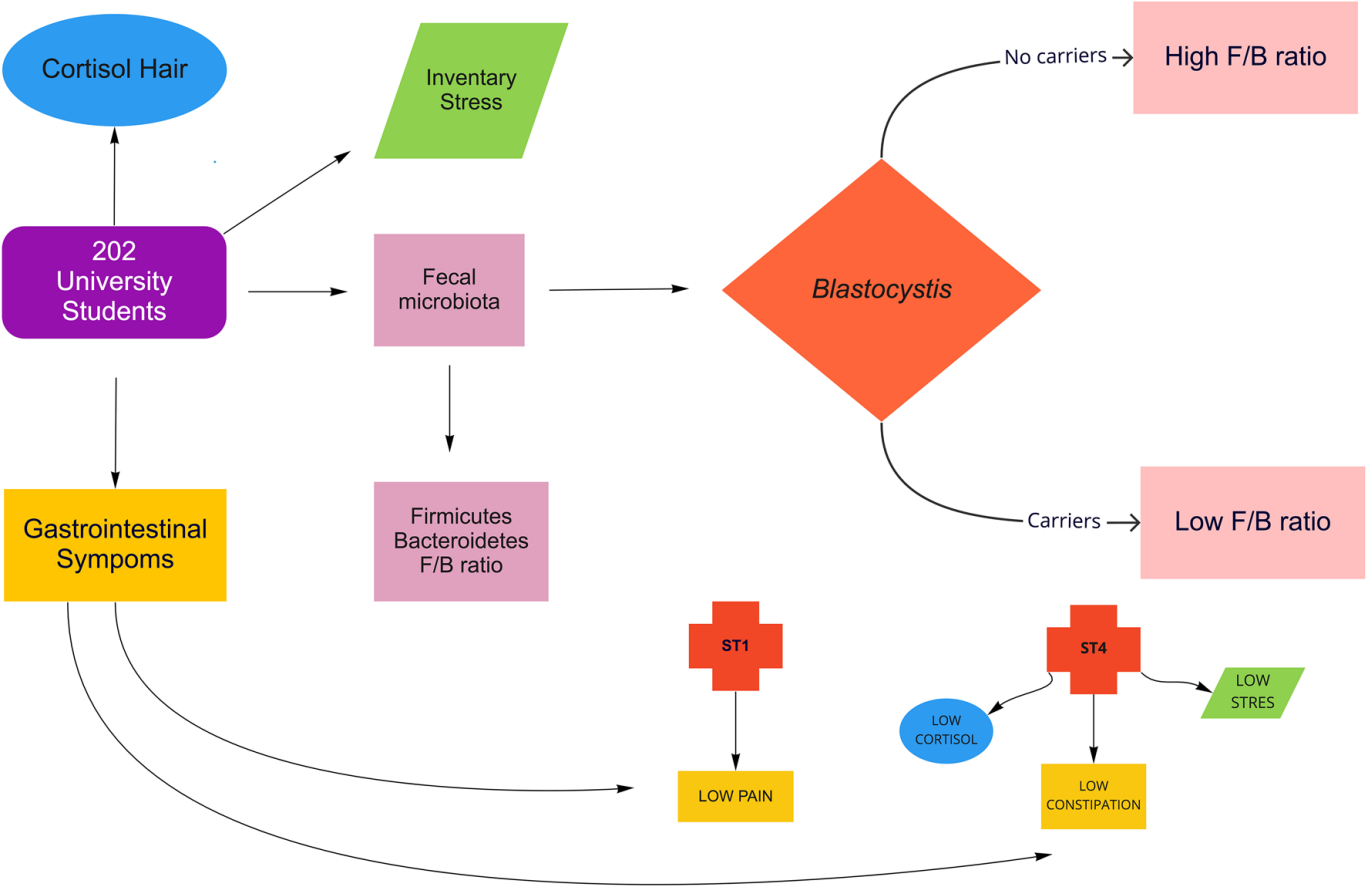
Relationship among *Blastocystis*, the *Firmicutes/Bacteroidetes* ratio and chronic stress in Mexican university students. Graphic Model. In this study we recruited two hundred university students; they were asked for a stool and hair sample to determine the fecal microbiota (*Firmicutes, Bacteroidetes*, *Firmicutes/ Bacteroidetes* ratio) *Blastocystis* subtypes 1,2,3,4,5 and 7) and cortisol in hair for the determination of chronic stress, respectively. Inventories of perceived stress and academic stress were applied, as well as questionnaires of gastrointestinal symptoms. Subjects not colonized by *Blastocystis* had a high *F/B* ratio, as opposed to subjects colonized by *Blastocystis*, who had a low *F/B* ratio. Regarding the subtypes, ST1 is related to less abdominal pain; subjects colonized by ST4 have lower cortisol levels, academic stress and perceived stress, and less constipation. ST1 (Subtype 1), ST4 (Subtype 4), *F/B* ratio (*Firmicutes/Bacteroidetes* ratio)

*Blastocystis* is a single-celled protist inhabitant of the gut microbiota found in both asymptomatic and symptomatic humans and animals [1]. The transmission is mainly fecal-oral, with a 1–60% human infection rate of, or 100% in some geographical regions [2]. *Blastocystis* is linked to irritable bowel syndrome (IBS), but its role in disease has been questioned considering its widespread nature [3]. Several cohort studies suggest a link between *Blastocystis* and IBS [4–6], while others do not [7, 8]. A possible explanation for these contradictory findings is the large genetic diversity observed within *Blastocystis* [9], in which some subtypes might indeed be linked to disease while others might not [10]. Currently, 22 subtypes have been identified, among which the first 17 have been recognized, and only ten have been identified in humans (ST1-ST9 and ST12) [11]. The subtypes ST1-ST4 are the most frequent, with a predominance of ST3 [2], which has been found most frequently in patients with clinical gastrointestinal and dermatological manifestations [12]. However, some studies suggest that the presence of *Blastocystis* increases the diversity of gut bacteria [13, 14], and healthier individuals often harbor a more significant gut microbiological diversity [15]. Because of this, it has been suggested that *Blastocystis* is a member of normal gut microbiota [16]. The gut microbiota contains more than 70% of all microorganisms in the human body [17], and it is involved in nutrition, regulation of immunity, and systemic inflammation. In the central system, the gut microbiota has been related to mood and behaviour regulation through the brain-gut-microbiota axis [17, 18], which is bidirectional, maintaining homeostasis in the central nervous system (CNS) and modulating gastrointestinal function [19]. Recently, the role of gut microbiota in stress has been evaluated. Stress processing occurs in the CNS through different pathways [20]. The brain´s communication to the intestine occurs through the autonomic nervous system (ANS) via the vagus nerve. This nerve regulates intestinal motility, bacterial colonization, and the size of the intestinal mucous layer [21], which provide information to the nodes of the enteric nervous system (ENS) with the consequent release of peptides and 5-hydroxytryptamine (5-HT) granules. The 5-HT is synthesized from tryptophan, which is regulated by the gut microbiota [22, 23]. The hypothalamus-pituitary-adrenal (HPA) axis works synergistically with the ANS resulting in the release of corticotropin-releasing hormone (CRH) from the paraventricular nucleus (NPV) into the portal circulation. The binding of CRH to the CRH receptor 1 (CRHR1) in the pituitary leads to Adrenocorticotropic hormone (ACTH) release. Systemic ACTH binds to the melanocortin type 2 receptor in the adrenal glands, resulting in de novo biosynthesis and release of glucocorticoids, within which cortisol is the main in humans [24]. In this context, the use of hair samples allows for retrospective measurement of steroid hormones, including cortisol as a chronic stress measuring, since this sample reflects the cortisol accumulation by the repeated and constant activation of the HPA axis [25, 26]. Furthermor1e of this measurement of stress, we used the Perceived Stress Scale (PSS), that is a psychological instrument for measuring the degree to which life situations are assessed as stressful [27], and the SISCO inventory of academic stress that recognizes the characteristics of stress that usually accompany students during their studies [28] (Fig. 1).

The relationship of *Blastocystis* with stress has not been widely studied. The microbiota may mediate the relationship with the parasite since colonization by *Blastocystis* has been associated with greater diversity and richness of bacteria. These characteristics have been considered as indicators of ecosystem stability and human health [13, 14]. In some studies, the presence of *Blastocystis* has been associated with a greater abundance of *Firmicutes* and other orders and genera, in contrast to *Bacteroidetes,* which are more abundant when this parasite is absent [8]. Besides, University students are a population subject to high psychological stress [29], which can be exacerbated by their poor lifestyle [30] and diet [31, 32]. For this reason, our objective was to analyze the relationship of colonization by *Blastocystis* and its subtypes 1–4 and 7 on the gut microbiota in university students by the *Firmicutes/Bacteroidetes* ratio, and on chronic stress by measuring academic stress, perceived stress, and cortisol levels.

## Materials and Methods

### Sample Collection

The study has a cross-sectional design; the sampling was non-probabilistic and was conducted between March and October 2018. University students of the medical surgeon and nutrition program were invited to participate. The study was approved by the Ethics Committee of the Faculty of Health Sciences from Universidad Juárez del Estado de Durango (PI-01–2018). The participants received information about the study and, subsequently, they signed an informed consent form. Later, they answered a digital format's gastrointestinal symptoms questionnaire, with of multiple-choice questions based on Rome III diagnostic criteria [33]. Exclusion criteria were the presence of chronic degenerative diseases and have received any pharmacological treatment and having dyed the hair in the last three months before the study.

### Measurement of Diet

Anthropometric characteristics of height and weight were recorded by a trained nutritionist. The students were questioned about their physical activity to proceed with the application through online platforms of a semi-quantitative food frequency questionnaire validated by Macedo et al. in 2013, which analyzes the average daily intake of food and nutrients in a year [34]**.**

### *Blastocystis* Identification

#### Parasitological Examination

Feces samples were collected in containers with 10% formaldehyde for coproparasitoscopic exams in triplicate. Each microscopic identification of *Blastocystis* was carried out on a different day of the deposition. A modified Ritchie technique was performed to prepare the samples to be observed later under the microscope with 10 × and 40 × objectives.

#### DNA Extraction

To confirm the microscopy diagnosis, Polymerase Chain Reaction (PCR) was used to detect *Blastocystis* and subtypes. A fresh sample was dispensed into a DNAse and RNAse-free sterile bottle and kept under refrigeration until transport to the laboratory, stored at − 20ºC until use. The extraction of nucleic acids from 200 mg of faeces was carried out using the E.Z.N.A.® Stool DNA Kit (USA). DNA concentration and purity were determined using NanoDrop 1000 Thermo Scientific (Saveen Werner ApS®, Denmark).

#### Determination of Genus Blastocystis by Polymerase Chain Reaction (PCR)

The extracted DNA samples were used to determine the presence of *Blastocystis* for Polymerase chain reaction (PCR). We used 3 μl of each DNA sample and Radiant™ Red 2 × Taqman Mastermix (Alkali Scientific Inc.) to a final volume of 13 μl for the PCR. The primers used were: F1- 5'-GGA GGT AGT GAC AATAAA TC-3' and R1- 5'-CGT TCA TGA TGA ACA ATT AC-3', which were constructed according to the partly published sequence of the Ribosomal 16-S like rRNA of *Blastocystis hominis* [35, 36] (T4 Oligo®, Irapuato, México).

#### Subtyping of *Blastocystis* using Sequence-Tagged Sites (STS) Primers

For the genotyping of *Blastocystis*, a set of sequence-tagged site primers derived from products of randomly amplified polymorphic DNA (RAPD) sequences were used [37, 38]. The primers used were SB83 (351 bp), SB155 (650 bp), SB227 (526 bp), SB332 (338 bp), SB340 (317 bp) and SB337 (487 bp) for subtypes 1, 2, 3, 4, 5 and 7, respectively (Table A1), according to a classification terminology [10]. Genotyping was performed from samples that were positive by PCR for *Blastocystis*. Four μl of each DNA sample positive for *Blastocystis* in a Polymerase chain reaction (PCR) was mixed with Radiant™ Red 2 × Taqman Mastermix (Alkali Scientific Inc.) with the primers described in Table A1 in a final volume of 13 μl.

**Table 1:** General characteristics and composition of the fecal microbiota of university students

Characteristic	Total	F/B ratio		
		High	Low	*p*
*n* (%)	202 (100)	102 (50.50)	100 (49.50)	
*Sex, n (%)*				
Man	68 (33.66)	38 (55.88)	30 (44.12)	0.275
Woman	134 (66.33)	64 (47.76)	70 (52.24)	
Age, *M* (RI)	20 (19–21)	20 (19–21)	20 (19–21)	0.247
*Residence, n (%)*				
Rural	30 (14.85)	17 (16.67)	13 (13)	0.464
Urban	172 (85.15)	85 (83.33)	87 (87)	
*Career, n (%)*				
Medicine	138 (68.32)	69 (50)	69 (50)	0.836
Nutrition	64 (31.68)	33 (51.56)	31 (48.44)	
*Physical activity, n (%)*				
Sedentary	82 (50.31)	47 (57.32)	35 (42.68)	0.201
Active	54 (33.13)	23 (42.59)	31 (57.41)	
Very active	27 (16.56)	12 (44.44)	15 (55.56)	
*Anthropomety, M (RI)*				
Height (m)	1.65 (1.59–1.72)	1.65 (1.59–1.72)	1.65 (1.58–1.72)	0.571
Wheight (kg)	68.35 (56.8–79.3)	68.45 (57.2–80.45)	67.2 (55.6–78.3)	0.777
BMI (kg/m2)	24.29 (21.08–28.76)	24.35 (21.46–28.82)	24.05 (21.02–28.5)	0.671
*Obesity, n (%)*				
Obese	38 (18.81)	47 (52.22)	43 (47.78)	0.660
Non obese	164 (81.19)	55 (49.11)	57 (50.89)	
*Abdominal pain, n (%)*				
Symptomatic	112(55.45)	57(50.89)	55(49.11)	0.900
Asymptomatic	90 (44.55)	45(50)	45 (50)	
*Stress markers*				
Hair cortisol (pg/mg), M (RI)	11.34 (7.66–16.58)	11.34(7.49–16.06)	11.37(7.84–16.89)	0.806
PSS score, Mean (± SD)	25.44 (± 8.10)	25.95 (± 7.69)	24.92 (± 8.49)	0.187
SISCO score, Mean (SD)	13.00 (± 4.54)	13.15 (± 4.52)	12.85 (± 4.58)	0.324
*Diet composition, M (RI)*				
Total energy (kcal)	2017.5 (1546.6–2528.6)	2071 (1534.2–2631)	1963.1 (1438–2498)	0.599
Fiber (g)	12 (7–18)	11 (7–18)	12 (7–21)	0.465
Carbohydrates (g)	242.5 (176–328.5)	245 (177–328)	218.5(171–349)	0.295
Proteins (g)	82 (64–116)	81 (64–122)	93 (59–112)	0.479
Lipids (g)	75.5 (47.5–106)	78 (49–110)	69 (47–94)	0.599

*BMI* body mass index, *m* meters, *m*^*2*^ square meters, *kg* kilograms, *RAU* relative abundance units, *F/B Firmicutes/Bacteroidetes* ratio, *PSSscore* perceived stress scale, *SISCOscore* SISCO academic stress inventory (psychological reactions), *kcal* kilocalories, *g* grams, *M* median, *RI* interquartile range, *SD* standard deviation. High *F/B* ratio > 0.83, Low *F/B* ratio < 0.83

The PCR conditions were: an initial denaturation step at 94 °C for four minutes; followed by 35 denaturation cycles at 94 °C for 30 s; annealing at 55 °C for 45 s; extension at 72 °C for 45 s; and a final extension at 72 °C for 10 min (PTC-100 thermocycler, MJ Research Inc) [36]. The ß-globin gene was amplified as an internal extraction control. The samples that were negative for gender, but beta-globin positive underwent subtyping. The PCR products were resolved in a 1.5% agarose gel (Ultrapure Agarose, Invitrogen™) stained with RedGel™ Nucleic Acid Gel Stain (Biotium), and a molecular weight marker was used to establish the size of the amplicon (100 pb DNA Ladder. Biobasic Inc.). Additionally, the samples were randomized to the analysis by PCR. Sanger sequencing was used to corroborate both the presence of *Blastocystis* and genotypes, contrasted them with sequences reported in https://blast.ncbi.nlm.nih.gov/Blast.cgi using a blast. The nucleotide sequences generated in the present study have been deposited in GenBank (https://www.ncbi.nlm.nih.gov/) under accession numbers: MZ351752-57.

### Identification of the *Firmicutes/Bacteroidetes*

The analysis of the microbiota profile was performed by real-time PCR (qPCR) using the 16S rRNA gene-specific and universal primers. The sequences of the primers were: *Bacteroidetes*: 798cfbB-AAACTCAAAKGAATTGACGG (Forward) and cfb967R-GGTAAGGTTCCTCGGCTAT (Reverse); *Firmicutes*: 928F-Firm-TGAAACTYAAGGAATTGACG (Forward) and 1040FirmR-ACCATGCACCACCTGTC (Reverse); universal: 926-F-AAACTCAAAKGAATTGACGG (Forward) and 1062R-CTCACRRCACGAGCTGAC (Reverse) [39] (T4 Oligo®, Irapuato, México).

To each PCR reaction, 5 μl of SYBR Green (Maxima SYBR Green qPCR Master Mix, Thermofisher Scientific TM), 1 μl of each primer (concentration of 5 pmol for Reverse and 10 pmol for Forward), 1 μl of DNA, and 2 μl DNAases and RNAases-free water were added to a final volume of 10 μl. Each reaction was performed in duplicate. The specificity of the amplification products and the absence of primer dimers were determined by performing melting curve analyses in all cases.

The analysis of the qPCR amplification was performed with the Rotor-Gene Q equipment (QIAGEN®, Germany). The samples were processed under the following amplification conditions: an initial thermal denaturation cycle of five minutes at 95 °C, alignment with 30 cycles at 59 °C for 15 s, and elongation for 20 s at 72 °C. The conditions were the same for the three pairs of primers used; Universal, *Bacteroidetes,* and *Firmicutes*. The results expression was carried out by quantifying the relative abundance units (RAU) of *Firmicutes* and *Bacteroidetes* with the formula RAU = 2^−∆Ct^, which: RAU = Relative Abundance Units and ∆Ct = Ct specific primers-Ct universal primers [40].

### Stress Measurement

#### Application of Stress Questionnaires

Two stress inventories were applied, the Perceived Stress Scale (PPS) and SISCO Inventory of academic stress. The PSS instrument was designed to measure the degree to which situations in life are valued as stressful, using the questionnaire in Spanish, previously validated by other research for this language [27]. The instrument consists of 14 items, all of which are self-applied and assess the level of perceived stress in the last month, with a 5-point response scale (0 = never, 1 = almost never, 2 = occasionally, 3 = often, 4 = very often). The total PPS score is obtained by inverting the scores of points 4, 5, 6, 7, 9, 10 and 13 (0 = 4, 1 = 3, 2 = 2, 3 = 1, and 4 = 0). A higher score indicates a higher level of perceived stress.

The Academic SISCO Inventory was designed to measure the amount of stress of academic origin perceived by students. This instrument comprises 31 items, with a filter item that, in dichotomous terms (yes–no), allows to determine if the respondent is a candidate or not to answer the inventory. The remaining items use a Lickert-type scaling of five categorical values (never, rarely, sometimes, almost always and always). The questionnaire is divided into three sections that identify the frequency in which the environment demands are valued as stressful stimuli, the frequency of symptoms or reactions to the stressful stimulus, and the frequency of use of coping strategies [28]. In this project, only the section questions on the frequency of symptoms or reactions to the stressor stimulus (psychological reactions questions) were used.

Before starting the project, a pilot study was carried out to validate the instruments on subjects from the same population (Faculty of Health Sciences). The Perceived Stress Scale (PSS) instrument was applied to 49 subjects, and the SISCO Inventory of academic stress (psychological reactions section) was completed only for 29 subjects, both by self-application in digital format. For the PSS instrument, a Cronbach alpha of 0.83 was obtained, reliability by test–retest analyzed by Pearson correlation was 0.7182. For the academic SISCO, a Cronbach alpha value of 0.9448 and reliability by test–retest analyzed by Pearson correlation of 0.6778 was obtained.

#### Extraction of Cortisol from Hair Samples

The hair samples were cut with surgical scissors at the level of the back of the head, as close to the base as possible. The first two centimeters (excluding the follicle) were selected for the sample. The samples were labelled and stored for later extraction and cortisol analysis by competitive enzyme-linked immunosorbent assay (ELISA) in pg/mg.

To process the sample, 15–20 mg of hair was taken, cut into 3–4 mm long pieces, and transferred to a 15 mL falcon tube to be washed with 2.5 mL isopropanol. Then, the tubes were centrifuged at 1800 rpm for three minutes, and the supernatant was decanted. The samples were left to dry at room temperature, and 1.5 mL of methanol was added, then sonicated for 30 min. The methanol was evaporated from the sample for four hours at 110 °C and reconstituted by adding 500 µL of PBS buffer (pH 7.4).

For cortisol ELISA determination, 50 µL of the standard solution was added per well, and 50 µL of the detection solution Ab was immediately added. The plate was covered and incubated for 45 min at 37 °C. The solution was decanted, and 300 µL of 1 × wash buffer was added. The procedure was repeated three times, and then 100 µL of HRP conjugate was added and incubated for 30 min at 37 °C. The solution was then decanted, and 300 µL of the 1 × wash buffer was added and repeated 5 times. Then 90 µL TBM was added and incubated for 15 min at 37 °C. Finally, 50 µl of the stop solution was added, and the absorbance was measured at 450 nm.

### Statistical Analysis

The Kolmogorov Smirnov normality test was used. Non-parametric U-Mann–Whitney tests were used for comparisons between the medians of two groups. The *Firmicutes/Bacteroidetes* ratio variable was operationalized by taking the cut-off point above and below the median of the RAU on each phylum. For the bivariate analysis, we used a chi-square test (*x*^2^) or Fisher’s exact test. The odds ratio (OR) and 95% confidence interval were estimated. A *P* value < 0.05 was considered significant. The statistical analysis was performed using the Stata® Statistics Package, version 14.0.

## Results

Thirty-six subjects were excluded because they presented insufficient samples and/or incomplete questionnaires, and a final sample of 202 participants was obtained: 68 men (33.66%) and 134 women (66.33%). The median age was 20 years (19–21), and only 18.81% presented obesity. The frequency of *Blastocystis* by microscopy was 51.98% and 52.97% by PCR. The fecal microbiota composition was obtained with a median of 0.801 (0.057–2.088) RAU of *Firmicutes*, and 0.82 (0.441–1.658) RAU of *Bacteroidetes.* The *Firmicutes/Bacteroidetes* ratio (F/B ratio) was 0.83 (Table 1).

No significant associations were obtained between the *F/B* ratio and the general characteristics of the students (Table 1); however, the *F/B* ratio was significantly associated with the colonization of *Blastocystis* (*p* =  < 0.0001). The 66.04% of the students with a low *F/B* ratio were colonized by *Blastocystis,* while in the students with a high F/B ratio, only 33.96% presented the parasite (Table 2).

**Table 2:** Composition of gut microbiota in relation to colonization by *Blastocystis*

*Blastocystis* *n* (%)	F/B ratio		*p*
	High	Low	
Colonized	36 (33.96)	70 (66.04)	< 0.0001*
Non colonized	64 (68.09)	30 (31.91)	

*F/B Firmicutes/Bacteroidetes,* High *F/B* ratio > 0.83; Low *F/B* ratio < 0.83; *p ≤ 0.05

The relationship of *Blastocystis* to diet composition (Table 3) was not significant, but students colonized by *Blastocystis* tended to consume more fiber (*p* = 0.116) and less protein (*p* = 0.099).

**Table 3:** Evaluation of dietary composition in relation to colonization by *Blastocystis*

Dietary composition *M* (RI)	*Blastocystis*		*p*
	Colonized	Non colonized	
Total energy (kcal)	1939.41 (1438–2631)	2047.97 (1561–2503)	0.681
Fiber (g)	13 (7–21)	10 (6–16)	0.116
Carbohydrates (g)	252(192–306)	228 (171–332)	0.780
Proteins (g)	75.5 (54–116)	88 (71–122)	0.099
Lipids (g)	68.5 (48–99)	78 (47–110)	0.700

*kcal* kilocalories, *g* grams, *M* medium, *RI* interquartile range

The analysis of the *Blastocystis* subtype showed that ST3 was the most prevalent in this group of students, with a total of 18 students. The relative abundance of *Firmicutes* and the *F/B* ratio were significantly lower in students colonized by ST1, 2, 4, and 7, except for ST3 (*p* = 0.120; *p* = 0.290) (Table 4). Only the ST4 showed a significant relationship with stress (Tables 4 and 5) since lower values of cortisol (*p* = 0.030) and lower scores on academic stress assessment (*p* = 0.040) were found.

**Table 4:** Composition of the gut microbiota, gastrointestinal manifestations, and hair cortisol in relation to the subtype of *Blastocystis*

Subtype	Fecal microbiota			Gastrointestinal manifestations				Hair cortisol (pg/mg)
	RAU *Firmicutes* *M* (RI)	RAU *Bacteroidetes* *M* (RI)	F/B ratio *M* (RI)	Abdominal pain		Constipation		
				Symptomatic *n* (%)	Asymptomatic *n* (%)	Symptomatic *n* (%)	Asymptomatic *n* (%)	M (RI)
ST1								
Positive	**0.0014** **(0.00003**–**0.37)**	0.87 (0.45–1.24)	**0.0031** **(0.0001**–**0.77)**	**2 (2.11)**	**11 (12.22)**	7 (6.14)	6 (8.45)	10.64 (4.63–14.38)
Negative	**0.87** **(0.16**–2.23)**	0.82 (0.42–1.79)	**0.91** **(0.108**–4.19)*	**93 (97.89)**	79 (87.78)*	107 (93.86)	65 (91.55)	11.37 (7.76 –16.74)
ST2								
Positive	**0.0014** **(0.00027**–**0.43)**	1.03 (0.73–1.68)	**0.0022** **(0.00021**–**0.74)**	5 (5.26)	3 (3.33)	6 (5.26)	2 (2.82)	13.77 (8.38–20.61)
Negative	**0.83** **(0.12**–2.19)*	0.81 (0.42–1.65)	**0.88** **(0.09**–3.74)*	90 (94.74)	87 (96.67)	108 (94.74)	69 (97.18)	11.34 (7.62–16.39)
ST3								
Positive	0.28 (0.04–0.89)	0.92 (0.49–1.24)	0.43 (0.08–2.22)	14 (14.74)	12 (13.33)	17 (14.91)	9 (12.68)	13.57 (9–17.8)
Negative	0.91 (0.57–2.19)	0.81 (0.38–1.77)	0.91 (0.071–3.63)	81 (85.26)	78 (86.67)	97 (85.09)	62 (87.32)	11.08 (7.4–16.58)
ST4								
Positive	**0.085** **(0.00009**–**0.5515)**	0.773 (0.3867–1.4383)	**0.047** **(0.00005**–**1.2518)**	6 (5.36)	10 (11.11)	**6** **(4.58)**	**10** **(14.08)**	**6.28** **(4.75**–**13.45)**
Negative	**0.874** **(0.145**–2.265)*	0.870 (0.4416–1.6586)	**0.892** **(0.1321**–4.2134)*	106 (94.64)	80 (88.89)	**125** **(95.42)**	**61** (85.92)*	**11.54** **(8.03**–16.73)*
ST7								
Positive	**0.00015** **(0.00003**–**0.95)**	0.75 (0.43–1.62)	**0.00013** **(0.000016**–**0.18)**	5 (5.26)	7 (7.78)	8 (7.02)	4 (5.63)	13.53 (7.95–18.76)
Negative	**0.86** **(0.16**–2.19)**	0.84 (0.44–1.65)	**0.91** **(0.13**–3.74)**	90 (94.74)	83 (92.22)	106 (92.98)	67 (94.37)	11.25 (7.66–16.58)

*RAU* relative abundance units, *F/B ratio Firmicutes/Bacteroidetes* ratio, *M* median, *RI* interquartile range, *n* number, % percentage, *pg* picograms, *mg* milligrams

*p ≤ 0.05; **p ≤ 0.001

**Table 5:** Evaluation of chronic stress inventories in relation to the subtype of *Blastocystis*

Subtype	Chronic stress			
	PSS score Mean (± SD)	*p*	SISCO score Mean (± SD)	*p*
ST1				
Positive	28.37 (± 8.36)	0.220	13.30 (± 5.96)	0.720
Negative	25.21 (± 8.06)		12.98 (± 4.44)	
ST2				
Positive	22.42 (± 11.17)	0.220	10.14 (± 2.19)	0.040*
Negative	25.55 (± 7.98)		13.11 (± 4.58)	
ST3				
Positive	24.10 (± 8.05)	0.360	12.5 (± 4.64)	0.970
Negative	25.66 (± 8.11)		13.09 (± 4.53)	
ST4				
Positive	23.5 (± 6.69)	0.150	10.87 (± 4.41)	0.025*
Negative	25.62 (± 8.62)		13.1 (± 4.52)	
ST7				
Positive	26.81 (± 6.85)	0.640	14.25 (± 5.59)	0.164
Negative	25.36 (± 8.17)		12.92 (± 4.47)	

*PSS score* perceived stress scale, *SISCO score* SISCO academic stress inventory (psychological reactions), *SD* standard deviation

^*^p ≤ 0.05

The gastrointestinal clinic was significantly related in a negative way to *Blastocystis* ST1 and ST4 (Table 4), since only two students with abdominal pain were colonized by ST1 (*p* = 0.007), and most of the students colonized by ST4 did not manifest constipation (95.42%) (*p* = 0.010).

## Discussion

We found a significant association between *Blastocystis* (subtypes 1, 2, 4, and 7) and the modification in the gut microbiota, which was determined as a low abundance of *Firmicutes* and a low *Firmicutes/Bacteroidetes* ratio (Tables 2 and 4).

It is not yet clear whether *Blastocystis* is a causal agent of altering the intestinal microbiota composition or if the metabolic and intestinal microbiota alterations provide the favourable conditions for its colonization. It has been suggested that a possible explanation for the presence of *Blastocystis* as a direct cause of dysbiosis is that bacteria are the main source of nutrition for this parasite [41]. Furthermore, *Blastocystis* can affect certain micro-organisms, as invasive as microbiota bacteria, through the induction of secretion of LL-37, a fragment of cathelicidin expressed in various immune cells, salivary glands, and in the epithelia of various organs in humans, including the digestive tract. It has been described the antimicrobial properties of LL-37 participating in the destruction of bacterial, prokaryotic, and fungal organisms by forming pores in the cell membrane and cell lysis [42]. It also enhances bacterial plasmid supply to intracellular Toll-Like Receptors (TLR’S), which induces a potent antibacterial response, or by indirect effects through the stimulation of immunity with activation in cellular chemotaxis and the production of pro-inflammatory cytokines by macrophages (TNFα) and monocytes (IL-1b) and anti-inflammatory cytokines (IL-10 and CCL3) [43, 44].

Conversely, the host can provide a suitable environment for colonization. This could be explained by the activity of the pyruvate ferredoxine oxidoreductase (PFO) and [FeFe] hydrogenase, enzymes essential in carbohydrate and hydrogen metabolism, respectively. These enzymes can be activated in the presence of bacterial products, being used as sources of nutrients for *Blastocystis*. However, the co-incubation of *Blastocystis* and intestinal commensal bacteria exhibit a mutualistic relationship, since a greater number of parasites and bacterial colony-forming units (CFUs) are evidenced, due they break down dead cells (including *Blastocystis*) to obtain a source of nutrients [16].

Also, in a healthy intestine, bacteria produce butyrate, the preferred metabolic substrate of colonocytes, as they use it in the mitochondrial oxidation pathway β that produces ATP. This pathway use molecular oxygen, leading to reduced oxygen concentration in the intestine, producing an environment suitable for strict anaerobes such as *Blastocystis* which could partly explain why this parasite is rare in patients with diseases related to intestinal dysbiosis [45]. It has been proposed that *Bacteroidetes* produce mainly acetate and propionate, while *Firmicutes* produce more butyrate [46]. The increase in *Firmicutes* would lead to increased butyrate production, which is contradictory to our results; therefore, it is speculated that in unhealthy subjects, butyrate-producing bacteria decrease and are replaced by other bacteria belonging to the same phylum [47].

In this study, colonization by ST4 was associated with a protective role in chronic stress, reflected by lower scores on the academic stress inventory and less cortisol in the hair, as well as less frequency of constipation and abdominal pain. ST1 colonization was also associated with the absence of abdominal pain (Table 4). Previously, ST1 and ST4 have been found more frequently in patients without irritable bowel syndrome (IBS) or asymptomatic patients [14, 48]. Also, the colonization with ST4 was associated with a low *F/B* ratio and reduced colonic hypersensitivity in rats, as well as it was founded a reduced expression of occludin and preservation of intestinal permeability [49]. Similarly, other studies have associated ST4 with a richer and more diverse microbiota, which would translate into a healthy microbiota [14, 50].

The relationship between ST4 and chronic stress and IBS can be indirect through the gut microbiota because of stress; since it has been reported the gut microbiota is an important component in the development of the nervous system and in the maintenance of its homeostasis [51]. Furthermore, gut microbiota regulates neuroplasticity, neurogenesis, and microglia activation in adult individuals [52]. Studies in germ-free rodents have demonstrated the microbiota's modulating effect on the stress response, as rodents lacking intestinal microbiota showed a more significant response to stressful stimuli [53]. Other studies in rodents have shown that certain probiotic species of microbiota decrease concentrations of CRH [54] and modulate GABA (probably via the vagus nerve), which are important pathways for regulation of the HPA axis [55].

In mammals, it has been studied the relationship between microbiota and hair cortisol concentration. In a population of children, a study of chronic psychosocial stress showed that the *Ruminococcaceae* family (phylum *Firmicutes*) correlated positively with hair cortisol concentrations [56]; while in squirrels, a positive correlation between hair cortisol concentrations and *Lachnospiraceae* (phylum *Firmicutes*) and negative with *Akkermansiaceae* (phylum *Verrucomicrobia*) was found [57]. However, we did not find an association between the abundance of *Firmicutes* and the *F/B* ratio with the markers of chronic stress (Table 1).

The present study gives a better understanding of some subtypes of *Blastocystis* on gut microbiota and chronic stress. However, we are aware of their limitations; only the most predominant phyla of intestinal microbiota were analyzed and not with more specific microbial taxa as in previous studies. Furthermore, including only healthy subjects in the study may determine the finding of gut microbiota with a low *Firmicutes/Bacteroidetes* ratio. Another limitation of the design is the sample size due to financial and time feasibility; however, more specific studies such as cases and controls and follow-up will be carried out for future studies.

This work has allowed us to integrate practically the different studies that have investigated the relationship of *Blastocystis* with the intestinal microbiota in an indirect way. In this study, we accomplished the main objective; however, further studies are required to fully understand the relationship between each subtype of *Blastocystis* with the *Firmicutes/Bacteroidetes* ratio and the perception of stress and cortisol levels in different study samples. Also, more detailed studies to determine the direct effects of *Blastocystis* on human gut microbiota diversity are necessary. The different pathogenic potential among the different subtypes, intrasubtypical variations [10], and the variable and non-specific modifications generated by *Blastocystis* subtypes among the species of each bacterial phylum, raise many questions to be investigated.

## Conclusions

Colonization by *Blastocystis *subtypes (ST1, 2, 4, and 7) modifies the gut microbiota at its two most representative phyla with a decrease in the *Firmicutes/Bacteroidetes* ratio in apparently healthy university students. Also, ST4 is probably implicated in a better response to chronic stress, suggesting that the presence of *Blastocystis* may serve as an indicator of homeostasis in the gut microbiota and the central nervous system.

## Supplementary Information

Below is the link to the electronic supplementary material.

Supplementary file 1 (DOCX 13 kb)
