# Supplementary material for: Relationship Among Blastocystis, the Firmicutes/Bacteroidetes Ratio and Chronic Stress in Mexican University Students
Source: Curr Microbiol. 2022 Jan 24;79(3):72. doi: 10.1007/s00284-021-02756-7 (PMC8784498; doi:10.1007/s00284-021-02756-7)
Supplement: Supplementary file 1 — (DOCX 13 kb) [file 284_2021_2756_MOESM1_ESM.docx]

**Table A1**

Different STS primer sets used for differential identification of *Blastocystis* subtypes.

| **Subtype** | **STS primer set** | **Sequences** | **GenBank accession no.** | **Product size** | **Clade in the SSU rRNA phylogenya** |
| --- | --- | --- | --- | --- | --- |
| I | SB83 | F-GAAGGACTCTCTGACGATGA  R-GTCCAAATGAAAGGCAGC | AF166086 | 351 | I |
| II | SB155 | F-ATCAGCCTACAATCTCCTC  R-ATCGCCACTTCTCCAAT | AF166087 | 650 | VII |
| III | SB227 | F-TAGGATTTGGTGTTTGGAGA  R-TTAGAAGTGAAGGAGATGGAAG | AF166088 | 526 | III |
| III | SB228 | F-GACTCCAGAAACTCGCA  R-TCTTGTTTCCCCAGTTATCC | AF166089 | 473 | III |
| III | SB229 | F-CACTGTGTCGTCATTGTTTTG  R-AGGGCTGCATAATAGAGTGC | AF166090 | 631 | III |
| IV | SB332 | F-GCATCCAGACTACTATCAACATT  R-CCATTTTCAGACAACCACTTA | AF166091 | 338 | VI |
| V | SB340 | F-TGTTCTTGTGTCTTCTCAGCTC  R-TTCTTTCACACTCCCGTCAT | AY048752 | 704 | II |
| VII | SB337 | F-GTCTTTCCCTGTCTATTCTGCA  R-AATTCGGTCTGCTTCTTCTG | AY048750 | 487 | IV |
